# Expression Profiling of LPS Responsive miRNA in Primary Human Macrophages

**DOI:** 10.4172/1948-5948.1000276

**Published:** 2016-03-10

**Authors:** Afsar Raza Naqvi, Sheng Zhong, Hong Dang, Jezrom B Fordham, Salvador Nares, Asma Khan

**Affiliations:** 1 Department of Periodontics, College of Dentistry, University of Illinois at Chicago, USA; 2 Department of Endodontics, Center for Pain Research and Innovation, School of Dentistry, University of North Carolina, USA; 3 Cystic Fibrosis/Pulmonary Research and Treatment Center, Marisco Lung Institute, University of North Carolina, USA

**Keywords:** microRNA, Lipopolysaccharide, Toll like receptor, Macrophages, Inflammation

## Abstract

microRNAs (miRNAs) have emerged as important regulators of the innate and adaptive immune response. The purpose of the present study was to interrogate miRNA profiles of primary human macrophages challenged with bacterial lipopolysaccharide (LPS) with focus on expression kinetics. We employed Nanostring platform to precisely characterize the changes in miRNA expression following different doses and durations of LPS exposure. Differentially expressed miRNAs were identified in response to LPS challenge with convergent and divergent expression profiles. Pathway analysis of LPS-responsive miRNAs revealed regulation of biological processes linked to key cell signaling (including PIK3-Akt, MAP kinase, ErbB) and pathogen response pathways. Our data provide a comprehensive miRNA profiling of human primary macrophages treated with LPS. These results show that bacterial Toll like receptor (TLR) ligands can temporally modulate macrophage miRNA expression.

## Introduction

The initial immune response to bacterial infection involves the recognition of conserved pathogen-associated molecular patterns (PAMPs) such as LPS, lipoteichoic acid and peptidoglycans by pathogen recognition receptors (PRRs) including toll-like receptors (TLRs), RIG-I-like receptors and NOD-like receptors [1–3]. PRRs are expressed by a variety of cell types including macrophages (Mφ), dendritic cells (DC), epithelial cells, fibroblasts and neutrophils [1,4]. Recognition of PAMPs initiates intracellular signaling cascades which results in the expression of inflammatory mediators with the aim of eliminating the invading microbes. Most microbial pathogens contain multiple PAMPs which are recognized by different PRRs. Furthermore, the same PAMP may activate different PRRs. Thus the host immune response has the ability to coordinate both cell-type specific as well pathogen–specific responses.

The human genome encodes 10 different TLRs (TLR1–10; 4,5). TLRs differ in their cellular localization, ligand specificity and adaptor molecules. TLR 1, 2, 4, 5 and 6 are present on the cell surface while, TLR 3, 7, 8, 9, and 10 are localized on endolysosomal membranes [4]. Each TLR can recognize specific ligands that will trigger unique immune response. Some TLRs can bind wide range of ligands (both foreign and host) but others have restricted ligand specificity (either foreign or host). For instance, TLR4 can bind LPS, heat shock proteins (HSPs), polysaccharides, viral proteins, etc., while TLR3 and TLR5 specifically recognizes double stranded RNA and bacterial flagellin, respectively [1,5]. Further, TLRs also differ in their requirement of adaptor molecule for signaling.

The outcome of TLR signaling is to prime cells for immune responses. This is primarily achieved by activation/repression of a large array of genes and microRNAs (miRNAs). The discovery of miRNAs has greatly expanded our understanding of the mechanisms that regulate gene expression. miRNAs are small (18–22 nucleotides), single stranded, noncoding RNA oligonucleotides. They regulate gene expression by binding to the 3’-UTR of target messenger RNAs (mRNAs) to down-regulate gene expression at the post-transcriptional level either by translational repression or mRNA degradation [6–8]. More than 2500 miRNAs are encoded in the human genome and they regulate a wide variety of biological processes including proliferation, differentiation, cell fate determination, apoptosis, signal transduction and others [6,7]. A growing body of evidence supports the key role of miRNAs in the activation of both the innate and adaptive immune response [9–11]. Studies have shown changes in miRNA expression following LPS stimulation in various types of immune cells viz., monocytes, macrophages, etc. Several early and late LPS responsive miRNAs have been reported including miR-146, miR-155, members of let-7 family, etc. These miRNAs serve as positive and negative regulators of TLR signaling. They achieve this by targeting the components of TLR signaling pathways. For instance, miR-146 downregulates TRAF6, IRAK1 and IRAK2 while, let-7i and miR-105 target TLR4 and TLR2, respectively [12–15]. Therefore, changes in miRNA expression are not only part of host response to LPS, but also function to precisely control this response. Thus, identification of miRNA repertoire responsive to LPS challenge will be crucial in various bacterial infections.

In this report, CD14+ human macrophages were challenged with TLR4 agonist *E. coli* LPS, a widely accepted model to examine the innate response to microbial infection [16], and miRNAs were measured by the Nanostring nCounter platform. NanoString nCounter has been shown to be a novel, effective alternative to SYBR green real-time PCR [17,18]. It provides discrete counts of RNA transcripts and is capable of a high level of precision and sensitivity at less than one transcript copy per cell. By providing discrete counts of RNA transcripts, the nCounter overcomes the saturation limitations of microarrays while avoiding the complex sequence analysis necessitated by RNA-Seq. The data gained provides precise and a real time changes in miRNA copy number and is highly relevant in clinical settings as demonstrated by various studies [19,20]. Here we demonstrate that miRNA expression in primary human macrophages is altered in time and dose-dependent manner upon *E.coli* LPS challenge. These miRNAs were predicted to target important signaling pathways.

## Materials and Methods

### Primary human macrophages culture and LPS stimulation

Freshly prepared buffy coats were collected from healthy donors (n = 3; Sylvan N. Goldman Oklahoma Blood Institute, Oklahoma City, OK) by density gradient centrifugation, as described earlier [21–25]. Briefly, PMBCs were purified using Ficoll Paque (GE Healthcare, Piscataway, NJ) based density centrifugation. PBMCs were incubated with magnetic-labeled CD14 beads (Miltenyi Biotech, Cologne, Germany), according to manufacturer’s instructions. The purity of CD14+ cells was 95%, as determined by flow cytometry. Monocytes were plated at 2 × 10^6^/ml in DMEM supplemented with penicillin (100 U/ml) and streptomycin (100 mg/ml). After 2 h, media was removed and replaced with media containing 10% FBS (Life Technologies, Grand Island, NY) and 50 ng/ml rhM-CSF (PeproTech, Rocky Hill, NJ) for Mφ differentiation. At day 7, cells were harvested, and surface expression of CD14, CD68, and HLA-DR was examined by flow cytometric analysis (data not shown). On day 7, primary Mφ were stimulated by *Escherichia coli* 055:B5 LPS (Sigma, St. Louis, MO) at 1 and 10 ng/ml and incubated for 1 and 8 hours. Controls consisted of differentiated Mφ cultured in the absence of LPS.

### Cell viability assay

Cell viability was determined using the CellTiter 96 AQueous Cell Proliferation Assay Kit (Promega, Madison, WI) as described by Naqvi et al. [25]. Briefly, LPS treated or untreated Mφ were incubated with MTS reagent for 2 h and absorbance at 490nm were collected on a 96-well plate reader (Victor^3^, Perkin Elmer, Waltham, Massachusetts, USA), according to manufacturer’s instructions.

### Total RNA preparation

At the end of the incubation period, cells were lysed and total RNA extracted using the miRNeasy Mini kit (Qiagen, Germantown, MD) according to manufacturer’s instructions. Total RNA was quantified and integrity assessed using the Nanodrop (Life Technologies) and Bioanalyzer (Agilent Technologies, Santa Clara, California, USA), respectively All RNA samples were processed by the Genomics and Bioinformatics Core, Lineberger Comprehensive Cancer Center at University of North Carolina at Chapel Hill.

### nCounter miRNA expression assay

miRNA profiling was performed using the Nanostring nCounter technology, a multiplexed, color-coded probe assay. One hundred nanograms of total RNA was used to generate cDNA libraries according to the manufacturer’s instructions. Ligation reactions, purification and dilution of probes were performed according to the manufacturer’s instructions (www.nanostring.com). Hybridization reactions were performed according to the manufacturer’s instructions with 5 ml of the five-fold diluted sample preparation reaction. All hybridization reactions were incubated at 65°C for a minimum of 18 h. Hybridized probes were purified and counted, following the manufacturer’s instructions. All samples were processed by the Genomics and Bioinformatics Core, Lineberger Comprehensive Cancer Center at University of North Carolina at Chapel Hill. Data Collection was carried out in the nCounter Digital Analyzer on the nCounter Prep Station and Digital Analyzer (NanoString Technologies, Seattle, WA, USA) at the Lineberger Comprehensive Cancer Center. For each assay, a high-density scan (600 fields of view) was performed. Digital images were processed and the barcode counts were tabulated in a comma separated value (CSV) format.

### Data analysis

Nanostring nCounter expression counts were inspected manually and samples with low counts throughout all probes comparable to the negative controls were identified as outliers and excluded from further analysis. The count data were normalized using the Bioconductor package, NanoStringNorm, using geometric means of low variance probes to adjust for sample content, and quantile normalization after background subtraction. The normalized expression values were log2 transformed and probes that do not have values greater than 4.587876 (or mean + 2 standard deviations of negative controls) in any of the samples were filtered out. Differential expression (DE) were analyzed by a 4-way ANOVA model with donor as random effect factor, LPS dose and time of treatment as fixed effect factors with dose and time interaction. The assay plate information was used as a surrogate for batch effect factor in the ANOVA model. Differentially expressed miRNAs were filtered at p-value < 0.05 and fold change > 1.5. DE miRNAs from different comparisons were pooled and the normalized log2 expression values were standardized across all DE miRNAs to mean of 0 and standard deviation of 1, before hierarchical clustering. The statistical analysis and clustering were performed using Partek Genomics Suite v6.6 (St. Louis, MS, USA)

### Prediction of miRNA targeted pathways

For each sample set, the LPS responsive miRNAs were uploaded onto DIANA-miRPath (http://diana.imis.athena-innovation.gr/DianaTools/index.php?r=mirpath/index) for pathway prediction. Since numerous predicted miRNA targets await validated, we selected DIANA-microT-CDS algorithm which included predicted miRNA targets.

## Results

### Macrophages respond to LPS by altering miRNA expression

**Figure 1:**
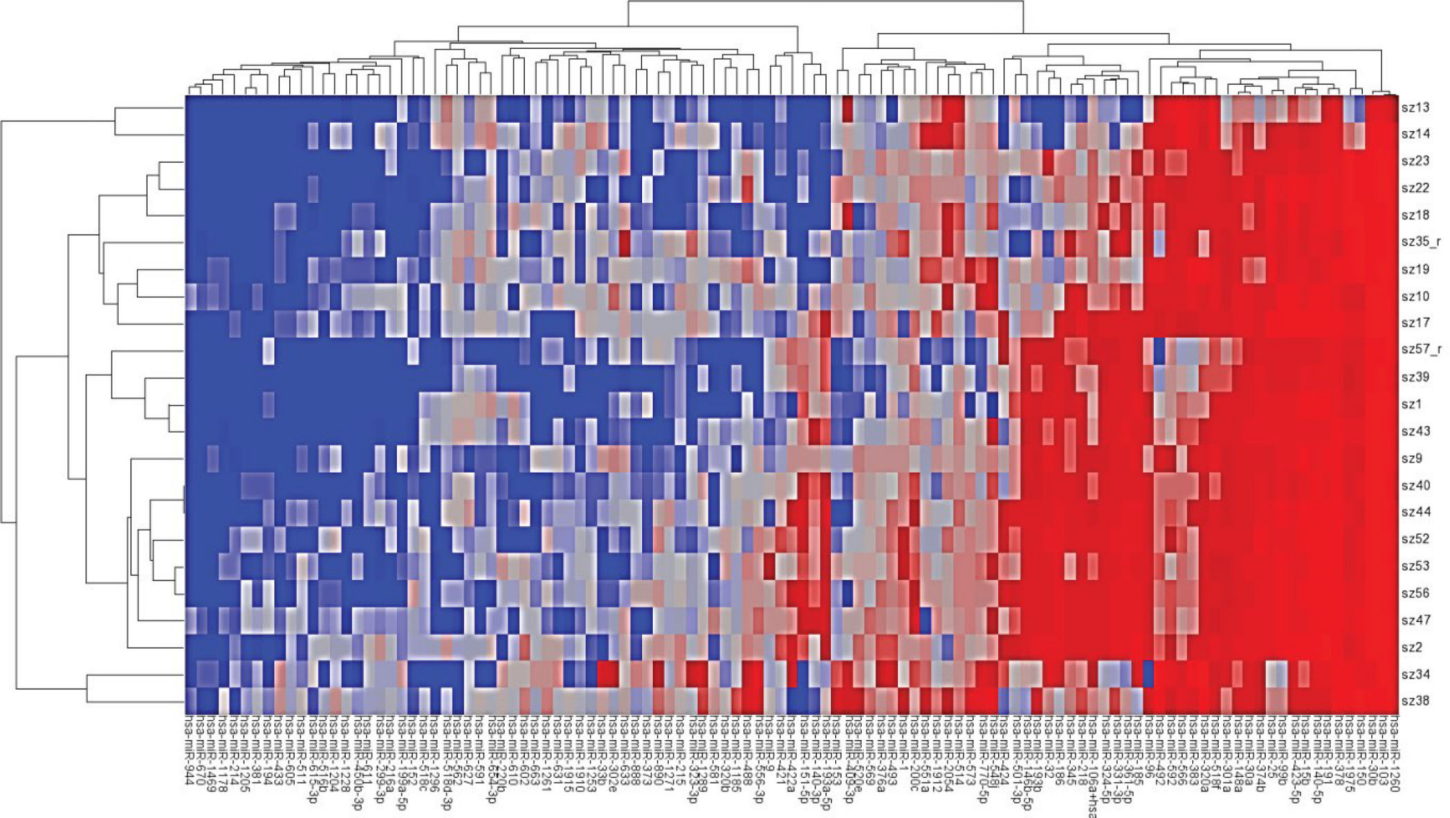
Heatmap showing miRNA expression in response to different doses and time points of LPS challenge. Samples are: Control#17,47,11; 1ng/ml;1hr#1,18,22,39,43; 1ng/ml;8hr#9,13,52,56; 10ng/ml;1hr# 2,19,23,40,44; 10ng/ml;8hr# 10,14,53,57.

**Figure 2:**
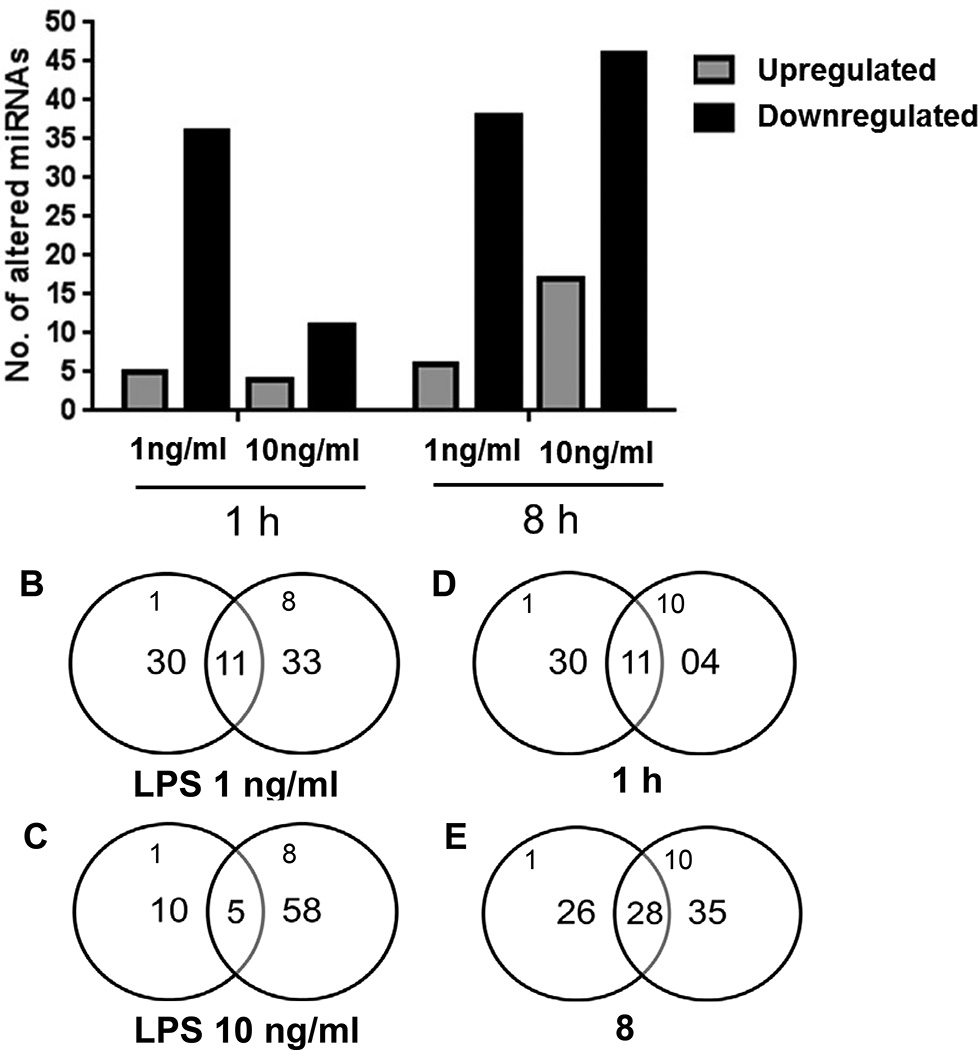
**(A):** Differential expression of miRNAs following LPS challenge at 1ng/ml and 10 ng/ml for 1 h or 8 hours. **(B–E):** Common sets of differentially expressed miRNA following LPS challenge at specific doses and time points.

Mφs respond to PAMPs by secreting proinflammatory cytokines that subsequently trigger both innate and adaptive arms of immunity. We first confirmed that 1ng/ml of LPS challenge is sufficient to induce secretion of proinflammatory cytokine TNFα (data not shown). We also confirmed that LPS treatment did not affect the cell viability. To examine the role of miRNAs in the early phases (1h and 8h) of LPS stimulation, we interrogated global miRNA profile in primary human Mφs. The time-kinetics of miRNA expression was analyzed by profiling 593 miRNAs using the Nanostring platform. We identified several miRNAs that were significantly altered upon LPS stimulation. Figure 1 shows a heat map of differentially expressed miRNAs across all samples. Interestingly, differentially expressed miRNAs were predominantly downregulated across all the dose and time points examined (Figure 2A). Mφs challenged with 1ng/ml *E. coli* LPS for 1 hour show altered expression of 41 miRNAs; of which 5 exhibit induced expression while 36 were downregulated (Figure 2A; Table 1A). At 8h, we noted 44 differentially expressed miRNAs compared with unchallenged controls (Figure 2A, Table 1B). Among these, 6 miRNAs exhibit increased expression while 38 were downregulated. We then searched for a common set of differentially expressed miRNA, and found 11 miRNAs that were common to both the 1h and 8h time points (Figure 2B, 2C). The expression patterns of 10 of these (miR-146b, miR-193a, miR-193b, miR-218, miR-424, miR-450b-3p, miR-511, miR-573, miR-605 and miR-890) were similar at both time points. Our profiling identified previously reported LPS responsive miRNAs including miR-125b, miR-146b, miR-200, 302a, and miR-511 [21–23]. Further, we also noticed altered expression of several miRNAs that were previously not reported including newly identified miRNAs such as miR-1178, miR-1261, miR-1912 and miR-2054, etc., (Tables 1A and 1B). These results show LPS stimulation modulates Mφ miRNA profiles temporally by altering expression of different subsets of miRNAs.

### Macrophages respond differentially to LPS dose

Next we compared the impact of LPS dose on Mφ miRNA profiles. To this end, Mφs were challenged with 1ng/ml and 10ng/ml of *E. coli* LPS and miRNA expression was monitored at 1 h and 8 h post incubation. Analysis of 1 h samples showed 41 (5 up and 36 down) differentially expressed miRNAs with a challenge dose of 1 ng/ml LPS while at 10 ng/ml we identified 15 miRNAs (4 up and 11 down) with significant change in expression (Figure 2A and Tables 1A and D). Among the differentially expressed miRNAs, 11 (miR-199a-5p, miR-218, miR-409a, miR-433, miR-511, miR-514, miR-551, miR-562, miR-556-3p, miR-1178 and miR-1205) were common to both doses (Figure 2D) and exhibited similar expression patterns (Tables 1A and C).

The impact of LPS dose was also analyzed at 8 h post incubation. In cells treated with 10 ng/ml LPS, we noticed 63 differentially expressed miRNAs among which 17 were upregulated and 46 were downregulated (Figure 2A). These results suggest that Mφ stimulated for longer time periods and with higher LPS dose exhibit significant changes in miRNAs profiles. Interestingly, we also noticed that the prolonged stimulation of Mφ induced expression of greater numbers of miRNAs at this challenge dose (63 vs 44). Comparing miRNA profiles of cells challenged with 1 and 10 ng/ml LPS for 8h we identified 28 miRNAs that were common to both doses (Figure 2E). Overall, our results indicate a correlation between miRNAs profiles and LPS dose in Mφ.

### LPS responsive miRNAs are predicted to target key signaling and pathogen recognition pathways

In order to investigate the impact of altered miRNA expression on biological output, we assessed the biological pathways and predicted targets of the LPS-responsive miRNAs. The miRNAs from each dataset were subjected to pathway analysis on DIANA miRPath v3.0 (http://snf-515788.vm.okeanos.grnet.gr/dianauniverse/index.php?r=mirpath). From this we developed a list of predicted targets and biological pathways for the differentially expressed miRNAs. The list of 15 relevant pathways with significant p-values (<0.01) for each dose and time response is provided in Table 2. Several signaling pathways namely PIK3-Akt, MAPK, ErbB, Wnt and TGF-β were highlighted. As expected, we noticed pathways involving pathogen recognition and clearance. These include endocytosis, bacterial invasion of cells and FcR-mediated phagocytosis. Polysaccharides and lipids metabolism, signaling and modification pathways were most commonly identified across all the datasets examined (Table 2A–D). These pathways are known to impact cell adhesion, motility, pathogen recognition and uptake.

Importantly, we observed a significant overlap in the pathways affected by miRNAs from different datasets indicating the role of miRNAs in polarizing the cellular responses. Nonetheless, analysis of miRNAs differentially expressed at higher LPS dose (10ng/ml) revealed pathways not shared with lower dose (1 ng/ml) challenge at similar time point. Among these are two key pathways that include phosphatidylinositol signaling system and bacterial invasion of cells. Taken together, these results show that miRNA modulation can affect various cellular processes.

## Discussion

The innate immune response to PAMPs is associated with modulation of the host cell transcriptome. LPS from various pathogens has been demonstrated to have profound impact on gene expression. Examining the regulation of such rapid changes in gene expression is key to elucidating the underlying mechanisms. miRNAs have emerged as potent regulators of mRNA stability and translation. Studies have shown involvement of several miRNAs in LPS treatment. However, the global miRNA profiles in primary human Mφ have not been previously comprehensively examined. Here we provide updated miRNA profiling of LPS-stimulated Mφs and quantitated miRNA expression changes as a function of time and challenge dose. Our data shows that LPS challenge alters the miRNA profiles of primary human Mφ and confirms differential expression of previously described miRNAs as well as miRNAs not reported in primary Mφ using the Nanostring platform including a common set of LPS-responsive miRNAs. As our profiling included miRNAs from recent miRBase database (version 17), several novel miRNAs with potential roles in LPS signaling and/or response are identified. The role of these newly discovered miRNA is yet to be understood. Nonetheless, the changes in their expression would undoubtedly have an impact on their potential targets. Investigations on elucidating the functional role of these miRNAs in LPS treatment will uncover finer details of LPS signaling.

We observed different sets of altered miRNAs in a time and dose dependent manner. For instance, only 11 miRNAs were common to 1 h and 8 h samples when Mφ were challenged with 1 ng/ml LPS (Figure 2A) while 28 were shared between 1 ng/ml and 10 ng/ml LPS at 8 hr (Figure 2C and D). Nonetheless, our pathway analysis revealed significant overlap of pathways affected by the miRNAs. For instance, PI3K-Akt pathway was commonly targeted by miRNAs. PI3K activation is key inhibitory pathway in limiting NFκB activity and therefore checks proinflammatory cytokine (e.g. TNF-α, IL-6) production following LPS stimulation [26–29]. Several LPS responsive miRNAs target same pathway indicating regulatory fine tuning of these critical signaling pathways. Of the top 20 pathways, we noticed ~15 that were common for all the datasets. While a single miRNA can control expression of hundreds of target mRNA, several miRNAs can also bind and cooperatively regulate expression of the same transcript [7,8]. Taken together, these observations suggest that similar cellular functions are regulated by different sets of miRNAs.

Global profiling identified several novel LPS responsive miRNAs and also confirms miRNAs that are previously demonstrated to respond and regulate LPS signaling. For instance, miRNA profiling of monocyte-derived Mφ and DCs identified miR-511 as being differentially induced [23]. Interestingly, overexpression of miR-511 was shown to increase levels of its validated target TLR4, thereby acting as positive regulator of TLR expression in DCs. In the present study, we show that miR-511 is downregulated in response to LPS and its expression varies with the dose. While miR-511 expression is ~12 fold downregulated in cells treated with 1ng/ml LPS, its expression is to ~5 folds at 10 ng/ml LPS dose. TLR4 expression is known to increase with LPS stimulation [23]. Taken together, these observations suggest that high LPS dose increases expression of miR-511 and which in turn, may contribute to elevated TLR4 levels.

We noted several miRNAs that were responsive to LPS from *E.coli* and other gram-negative bacteria including the periodontal pathogens *A. actinomycetemcomitans* and *P. gingivalis* derived LPS [21]. While *E.coli* LPS is TLR4 agonist, *A. actinomycetemcomitans* and *P. gingivalis* LPS are TLR2 and TLR2/4 agonist, respectively. This indicates that LPS signaling emanating from different TLRs converge at the activation of common set of transcription factors regulating expression of these miRNAs. Our recent findings showed that miR-24 and miR-30 regulate phagocytosis of *E. coli* by myeloid inflammatory cells [25]. Both miR-24 and members of miR-30 were downregulated in *E. coli* LPS treated Mφ and this corroborates with our findings showing reduced expression of these miRNAs in *E.coli* challenged myeloid cells. Together, these observations indicate that highly similar impact on miRNA expression by *E. coli* LPS as well as whole bacteria which will have functional implication on downstream functions. Consistent with this, we noted that LPS responsive differentially expressed miRNAs were predicted to target pathways involved in pathogen recognition and uptake.

TLR4 signaling can be activated by either MyD (MyD-dependent) or TRIF (MyD–independent) adaptor proteins. MyD-dependent signaling culminates in the activation of NFκB and MAPK (specifically p38 or JNK) which induce expression of early response genes including proinflammatory cytokines [1,2,4]. On the other hand, TRIF adaptor leads to the activation of IRF3 that induces IFN-β and interferon inducible genes. Pathway analyses of deregulated miRNAs highlight several cell signaling pathways that are integral to myeloid cell-mediated innate responses. Our *in silico* analysis highlight MAPK, Ras, Raf1 signaling to be impacted by LPS responsive miRNAs. PI3K-Akt pathway/phosphatidylinositol signaling was significantly impacted by differentially expressed miRNAs across all the datasets as predicted in our analysis. Akt-1 has been demonstrated to play key role in endotoxin tolerance [22]. This protein regulates expression of various miRNAs including let-7e and miR-181c (positively) and miR-155 and miR-125b (negatively). Let-7e controls TLR response through targeting of TLR4 while, miR-155 regulates tolerance to immune response by targeting suppressor of cytokine signaling 1 (SOCS1). Evidently, Akt −/− mice fails to exhibit endotoxin resistance suggesting the key role of Akt pathway. This further confirms that macrophages respond to *E. coli* LPS challenge by altering expression of miRNAs that regulate both MyD-dependent and independent signaling cascades and can control signaling cascades leading to their own expression through feedback mechanisms.

Innate immunity constitutes the first line of defense by which the host recognizes and responds to invading pathogens or their conserved molecular patterns. Indeed, defective TLR signaling has been associated with plethora of pathophysiological conditions and diseases. Single nucleotide polymorphisms (SNPs) in TLR2 and TLR4 have been associated with childhood asthma, chronic obstructive pulmonary disease (COPD) and colorectal cancer [30–32]. Increased TLR4 expression leads to type 2 diabetes by reducing insulin secretion by β-cells and together with TLR2 and TLR9 contribute to the development of neurological disorders like schizophrenia and autism [32,33]. These studies highlight importance of TLRs in immune response and maintaining human physiology.

In summary, our results demonstrate that primary human macrophages respond to the TLR4 agonist, *E. coli* LPS by modulating expression of various miRNAs. These miRNA profiles are responsive to LPS dose and incubation time. In turn, these miRNAs are predicted to target key genes linked to immunity, inflammation and pain. This indicates that miRNAs play an important role in shaping innate immune responses by Mφ.

## Figures and Tables

**Table 1:** List of differentially expressed miRNAs responsive to *E. coli* LPS dose and time identified in primary human macrophages.

A. Human macrophages were treated with 1ng/ml E.coli LPS for 1 hour.		
miRNA	Fold change	p-value
hsa-miR-556-3p	−31.2343	0.000558
hsa-miR-890	−27.2135	3.06E-05
hsa-miR-199a-5p	−21.3307	0.002843
hsa-miR-1204	−13.9828	0.03219
hsa-miR-488	−12.1633	0.02769
hsa-miR-511	−11.7723	0.010604
hsa-miR-302e	−11.3793	0.00766
hsa-miR-216a	−10.741	0.033464
hsa-miR-516b	−10.4053	0.015952
hsa-miR-409-3p	−10.0526	0.023857
hsa-miR-1261	−9.57106	0.009325
hsa-miR-376a	−8.6461	0.003217
hsa-miR-33b	−8.18745	0.010264
hsa-miR-1253	−7.87889	0.037582
hsa-miR-2052	−7.77889	0.045247
hsa-miR-633	−7.76623	0.021612
hsa-miR-605	−7.68153	0.029125
hsa-miR-424	−7.43781	0.020846
hsa-miR-770-5p	−7.01844	0.011542
hsa-miR-1205	−6.95321	0.012067
hsa-miR-194	−6.89172	0.032392
hsa-miR-569	−6.82692	0.025647
hsa-miR-450b-3p	−6.64106	0.030425
hsa-miR-337-3p	−6.51625	0.043579
hsa-miR-548j	−6.47611	0.039445
hsa-miR-1912	−6.17196	0.032997
hsa-miR-433	−5.66386	0.031759
hsa-miR-1271	−5.38944	0.004058
hsa-miR-193b	−5.17428	0.045688
hsa-miR-1178	−4.85041	0.018656
hsa-miR-146b-5p	−4.62378	0.046233
hsa-miR-218	−4.59623	0.035773
hsa-miR-320a	−3.59125	0.020025
hsa-miR-193a-5p	−3.5568	0.035694
hsa-miR-106a/17	−3.24695	0.016031
hsa-miR-573	−2.80172	0.045195
hsa-miR-514	2.50392	0.047209
hsa-miR-562	7.51529	0.025695
hsa-miR-591	9.93073	0.026911
hsa-miR-551a	11.1935	0.000599
hsa-miR-1296	17.3165	0.02919

B. Human macrophages treated with 1 ng/ml E. coli LPS for 8 hours.		
miRNA	Fold change	p-value
hsa-miR-193a-5p	−28.1509	0.000100164
hsa-miR-424	−26.3899	0.0018661
hsa-miR-602	−23.9685	0.0288658
hsa-miR-186	−23.6563	0.0192091
hsa-miR-361-5p	−22.2274	0.0156237
hsa-miR-218	−21.2598	0.000968802
hsa-miR-193b	−17.0989	0.00420067
hsa-miR-185	−17.0196	0.0349622
hsa-miR-373	−14.6641	0.0415649
hsa-miR-605	−14.0025	0.0125141
hsa-miR-125b	−13.5135	0.0196724
hsa-miR-150	−13.2099	0.00240754
hsa-miR-331-3p	−13.0433	0.00284728
hsa-miR-196b	−11.6311	0.0415904
hsa-miR-488	−11.4972	0.0447984
hsa-miR-151-5p	−11.2514	0.0110432
hsa-miR-140-3p	−10.0222	0.0224999
hsa-miR-32	−9.82883	0.0413303
hsa-miR-324-5p	−9.69977	0.00215447
hsa-miR-1975	−9.63392	0.00459007
hsa-miR-450b-3p	−9.32785	0.0212821
hsa-miR-511	−9.04912	0.0294384
hsa-miR-532-3p	−8.28998	0.0490303
hsa-miR-615-3p	−8.2303	0.0262171
hsa-miR-493	−7.40798	0.0359485
hsa-miR-890	−6.87393	0.00475192
hsa-miR-301a	−6.71513	0.0211866
hsa-miR-96	−6.65422	0.045645
hsa-miR-146b-5p	−6.54631	0.0281515
hsa-miR-374b	−5.29633	0.0186839
hsa-miR-99b	−4.58917	0.0454594
hsa-miR-345	−4.21347	0.0418053
hsa-miR-15b	−3.73893	0.00777077
hsa-miR-423-5p	−2.58407	0.044588
hsa-miR-200c	−2.50166	0.0441161
hsa-miR-1274b	−2.14825	0.00956674
hsa-miR-221	−2.13986	0.00381351
hsa-miR-24	−1.74958	0.020932
hsa-miR-514	2.88047	0.0379482
hsa-miR-573	3.10818	0.0438976
hsa-miR-518f	3.30254	0.0304381

C. Human macrophages treated with 10ng/ml E. coli LPS for 1 hour.		
miRNA	Fold change	p-value
hsa-miR-581	−17.511	0.045932
hsa-miR-199a-5p	−9.06846	0.019608
hsa-miR-409-3p	−7.46168	0.043875
hsa-miR-153	−6.914	0.041517
hsa-miR-518c	−5.97653	0.025675
hsa-miR-511	−5.88614	0.050505
hsa-miR-1205	−5.26955	0.026363
hsa-miR-556-3p	−5.16818	0.04638
hsa-miR-433	−4.86279	0.046782
hsa-miR-1178	−4.57924	0.022404
hsa-miR-218	−4.09269	0.049502
hsa-miR-514	2.81549	0.028259
hsa-miR-562	9.75423	0.013981
hsa-miR-518d-3p	11.0995	0.025983
hsa-miR-551a	13.2433	0.000346

D. Human macrophages treated with 10ng/ml E. coli LPS for 8 hour.		
miRNA	Fold change	p-value
hsa-miR-186	0.00212131	−95.7265
hsa-miR-424	0.000360098	−57.373
hsa-miR-29b	0.00793683	−45.433
hsa-miR-27a	0.0194383	−40.4845
hsa-miR-32	0.00307869	−40.1821
hsa-miR-193a-5p	4.56E-05	−37.9784
hsa-miR-890	5.02E-05	−30.7255
hsa-miR-891a	0.0294375	−30.0668
hsa-miR-193b	0.00131651	−28.6328
hsa-miR-151-5p	0.00155507	−26.6324
hsa-miR-331-3p	0.000649021	−23.0796
hsa-miR-19a	0.0250877	−22.7717
hsa-miR-532-3p	0.00725674	−22.5814
hsa-miR-185	0.0238436	−21.8341
hsa-miR-18b	0.0138967	−14.7544
hsa-miR-423-3p	0.0183292	−13.8494
hsa-miR-140-3p	0.0127852	−13.1113
hsa-miR-218	0.005384	−10.9014
hsa-miR-574-3p	0.0178887	−10.8706
hsa-miR-324-5p	0.00167955	−10.5147
hsa-miR-520d-5p+miR- 527+miR-518a-5p	0.0332644	−10.447
hsa-miR-301a	0.00822971	−9.68532
hsa-miR-605	0.0279584	−9.47062
hsa-miR-516a-5p	0.0308221	−9.39405
hsa-miR-496	0.0110786	−9.2882
hsa-miR-31	0.0086838	−9.28772
hsa-miR-150	0.0071677	−8.85345
hsa-miR-146b-5p	0.0164869	−8.14325
hsa-miR-433	0.0299136	−6.82542
hsa-miR-1975	0.0143885	−6.44715
hsa-miR-107	0.0280177	−5.66795
hsa-miR-1205	0.0420576	−5.11632
hsa-miR-106a/17	0.00522892	−4.78058
hsa-miR-188-5p	0.0445391	−4.3485
hsa-miR-374b	0.0351116	−4.29073
hsa-miR-15b	0.00462133	−4.19912
hsa-miR-345	0.0434297	−4.15768
hsa-miR-376a	0.0476912	−4.12547
hsa-miR-532-5p	0.027525	−3.65767
hsa-miR-509-3p	0.0326475	−3.38043
hsa-miR-1260	0.0254564	−2.94232
hsa-miR-30c	0.0451624	−2.83118
hsa-miR-22	0.0228818	−2.76943
hsa-let-7a	0.0243692	−2.443
hsa-miR-221	0.00143387	−2.40003
hsa-miR-1274b	0.008082	−2.19725
hsa-miR-764	0.0468539	3.29166
hsa-miR-573	0.0348119	3.31514
hsa-miR-450b-5p	0.0399014	3.68501
hsa-miR-1180	0.0134275	3.71005
hsa-miR-383	0.00835454	3.82831
hsa-miR-760	0.0359474	4.69097
hsa-miR-449a	0.0445483	4.86847
hsa-miR-759	0.045053	4.934
hsa-miR-566	0.0181896	5.16446
hsa-miR-2054	0.0210186	7.09867
hsa-miR-432	0.0379926	7.3013
hsa-miR-551a	0.00374997	7.78185
hsa-miR-631	0.0415932	12.3371
hsa-miR-924	0.0284851	13.7512
hsa-miR-644	0.0108704	17.7977
hsa-miR-1323	0.00598167	21.0441
hsa-miR-1915	0.00463882	21.5772

**Table 2:** List of pathways predicted to be targeted by LPS responsive miRNAs. Only top 15 pathways are mentioned.

A. Predicted pathways of Mφ miRNAs responsive to 1ng/ml *E. coli* LPS challenged for 1 h.			
KEGG pathway	p-value	#genes	#miRNAs
Adherens junction	9.68E-12	59	30
Cell cycle	6.11E-06	82	31
TGF-beta signaling pathway	6.67E-05	51	30
ErbB signaling pathway	0.000115	59	28
Endocytosis	0.000192	123	31
Focal adhesion	0.000192	129	32
p53 signaling pathway	0.000258	50	27
Rap1 signaling pathway	0.000259	127	30
PI3K-Akt signaling pathway	0.000521	198	31
Regulation of actin cytoskeleton	0.002879	124	29
mTOR signaling pathway	0.003035	41	27
AMPK signaling pathway	0.003339	78	29
ECM-receptor interaction	0.006131	46	28
Fc gamma R-mediated phagocytosis	0.035122	55	25
Ras signaling pathway	0.035122	123	30

B. Predicted pathways of Mφ miRNAs responsive to 1ng/ml *E. coli* LPS challenged for 8 h.			
KEGG pathway	p-value	#genes	#miRNAs
ErbB signaling pathway	5.59E-06	63	32
TGF-beta signaling pathway	5.74E-06	53	31
Endocytosis	2.01E-05	130	32
Ras signaling pathway	0.000149	133	35
MAPK signaling pathway	0.000745	151	37
Wnt signaling pathway	0.001299	86	33
Regulation of actin cytoskeleton	0.002783	125	32
Rap1 signaling pathway	0.002783	124	34
Adherens junction	0.014892	47	31
Focal adhesion	0.014892	117	33
cGMP-PKG signaling pathway	0.027851	93	34
AMPK signaling pathway	0.027851	70	31
PI3K-Akt signaling pathway	0.027851	182	34
SNARE interactions in vesicular transport	0.029446	21	22
p53 signaling pathway	0.039423	42	26

C. Predicted pathways of Mφ miRNAs responsive to 10 ng/ml *E. coli* LPS challenged for 1 h.			
KEGG pathway	p-value	#genes	#miRNAs
AMPK signaling pathway	7.67E-05	42	10
mTOR signaling pathway	0.000919	24	10
Phosphatidylinositol signaling system	0.001834	24	9
TGF-beta signaling pathway	0.008479	23	10
N-Glycan biosynthesis	0.005766	12	8
Adipocytokine signaling pathway	0.019586	22	9
mRNA surveillance pathway	0.020065	28	11
RNA degradation	0.036259	27	10
Glycosylphosphatidylinositol(GPI)-anchor biosynthesis	0.03644	9	6
Wnt signaling pathway	0.03644	39	11
Gap junction	0.037595	21	11
cGMP-PKG signaling pathway	0.046164	44	10
Sphingolipid signaling pathway	0.048088	31	10
Glycosaminoglycan biosynthesis - keratan sulfate	0.048206	4	3

D. Predicted pathways of Mφ miRNAs responsive to 10 ng/ml *E. coli* LPS challenged for 8 h.			
KEGG pathway	p-value	#genes	#miRNAs
ErbB signaling pathway	1.35E-07	73	44
ECM-receptor interaction	4.50E-06	55	39
Ras signaling pathway	9.94E-06	154	49
Focal adhesion	2.26E-05	148	48
Endocytosis	6.10E-05	147	47
TGF-beta signaling pathway	0.000179	55	38
Mucin type O-Glycan biosynthesis	0.000251	20	23
Rap1 signaling pathway	0.000202	145	48
Adherens junction	0.002025	56	43
Wnt signaling pathway	0.000226	100	46
Viral carcinogenesis	0.001341	118	47
Phosphatidylinositol signaling system	0.002201	56	43
Bacterial invasion of epithelial cells	0.00222	56	39
PI3K-Akt signaling pathway	0.002786	217	48
MAPK signaling pathway	0.002989	169	50
